# Supplementary material for: Unsupervised Learning for the Automatic Counting of Grains in Nanocrystals and Image Segmentation at the Atomic Resolution
Source: Nanomaterials (Basel). 2024 Oct 10;14(20):1614. doi: 10.3390/nano14201614 (PMC11510824; doi:10.3390/nano14201614)
Supplement: Supplementary file 1 [file nanomaterials-14-01614-s001.zip › nanomaterials-3249567-supplementary.pdf]

Supporting Information

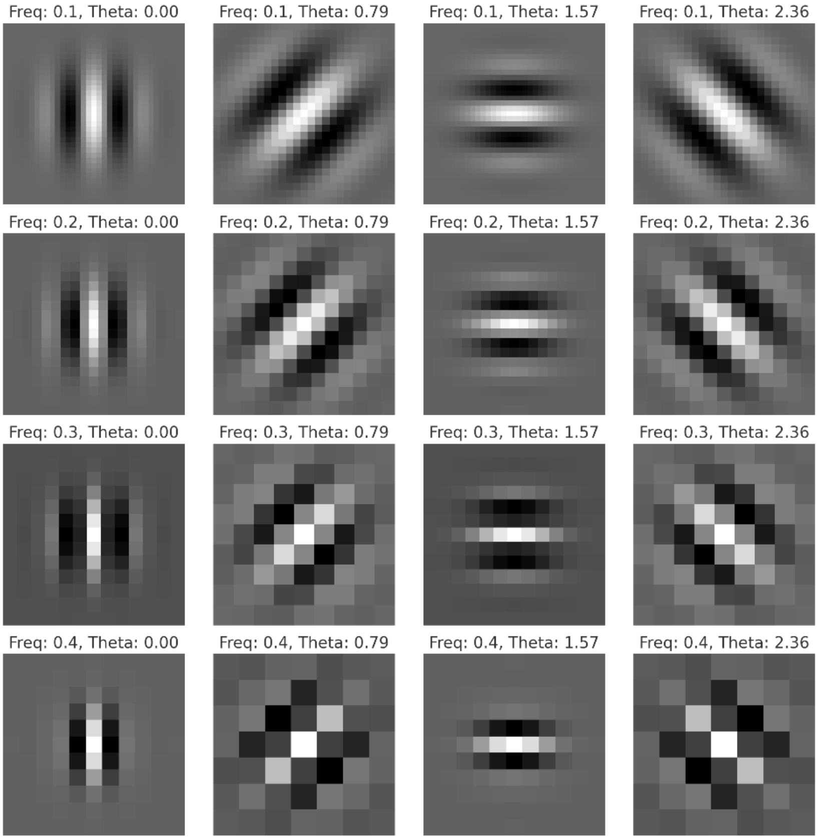

Figure S1. Visualization of the real part of a Gabor filter for different wavelength and orientations.

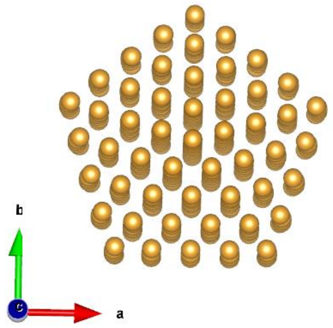

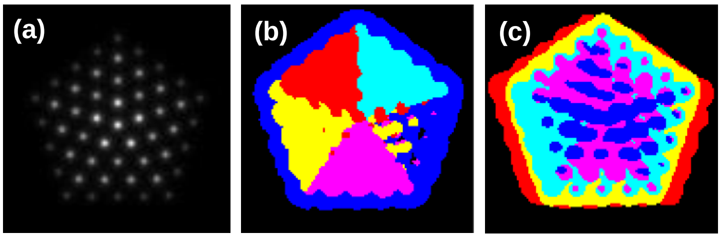

Figure S3. Segmentation of nanoparticles with various clustering methods. The number of clusters is 6. (a) Simulated HAADF STEM image of the Au nanoparticle. Segmentation result with (b) NMF followed by K--medians method, (c) K-means method without NMF process.

Figure S2. Atomic model of the Au nanoparticle with five fold twins.

	Ground truth	k=2	k=4	k=6	k=8
CORREL	1	0.82	0.96	1	0.97
CHISQR	0	2.96	0.64	0.06	0.3
INTERSECT	1	0.61	0.79	0.91	0.76
BHATTACHARYYA	0	0.51	0.34	0.12	0.34

Table S1. Image similarity test result. Ground truth is set for criteria. In case of CORREL and INTERSECT, the image shows the best similarity if the value is closest to 1. However,, in the case of CHISQR and BHATTACHARYYA, the image shows the best similarity if the value is closest to 0.

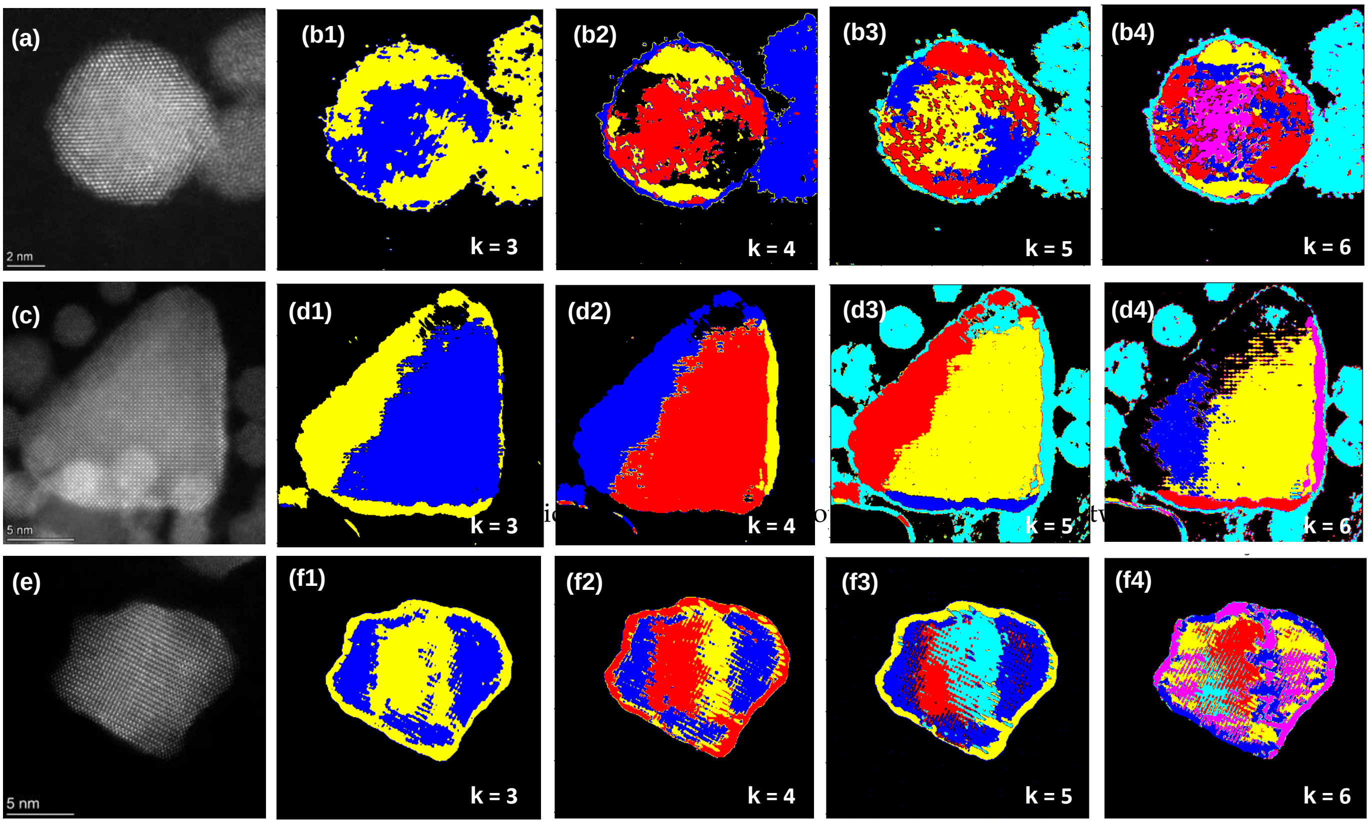

Figure S4. (a), (c), (e) Complete field of view of the micrograph containing the intermetallic nanoparticle and amorphous nanoparticles. (b1-4), (d1-4), (f1-4) segmented images with various k values

To show the computational complexity, psudocode schematization is included.

Pseudocode Schematization

```
// Comprehensive Image Processing and Clustering Pipeline

1. Gabor Filter Application:
   a. Load Initial Image:
      - Read image 'Particleimage.png' from 'trainingimages/Image/' directory
      - Convert image to grayscale
      - Resize image to 25% of original size

   b. Set Up Gabor Filter:
      - Define save directory as 'trainingimages/Image'
      - Create Gabor filter array with:
        - Wavelengths: [4, 4.5, 5, 5.5, 6, 6.5, 7]
        - Orientations: [0, 5, 10, ..., 170, 175] (36 values in 5-degree increments)

   c. Apply Gabor Filter:
      - Apply Gabor filter array to the image
      - Store results in gaborMag

   d. Process and Save Filtered Images:
      - For each filter in the Gabor array (252 total):
        - Scale the filtered image (multiply by 0.001)
        - Save the filtered image as 'filtered_image_X.png' (X is filter index)

2. Feature Extraction from Filtered Images:
   a. Set folder_path to Filtered Image'
   b. Get list of .png files in folder
   c. Initialize feature_matrix with dimensions (num_images, 512*512)
   d. For each image file:
      - Read grayscale image
      - Flatten image into 1D array
      - Store flattened array as row in feature_matrix

3. Clustering and Merging Process:
   a. Normalize feature_matrix (divide by 255.0)
   b. Set distance_threshold and angle_threshold
   c. Define merge_clusters function:
      - Calculate pairwise distances and angles between centroids
      - Merge clusters based on thresholds
      - Reassign labels
   d. For n_clusters from 2 to 10+alpha:
      - Perform NMF (Non-negative Matrix Factorization):
        - Set n_components = n_clusters
        - Apply NMF to normalized feature_matrix
      - Normalize NMF components (H matrix)
```

- Apply K-means clustering on normalized H matrix
- Merge clusters using merge_clusters function
- Save final labels to text file

4. Visualization:
    a. Reshape final labels to 1x512x512
    b. Define custom color map
    c. Create image plot using final labels and custom color map
    d. Add color bar and title
    e. Display plot

// Helper Functions

merge_clusters(centroids, labels, distance_threshold, angle_threshold):
- Calculate pairwise distances and angles between centroids
- For each pair of centroids:
    - If distance < distance_threshold and angle < angle_threshold:
        - Merge clusters
- Reassign labels to merged clusters
- Return new labels
